# Musical auditory stimulus acutely influences heart rate dynamic responses to medication in subjects with well-controlled hypertension

**DOI:** 10.1038/s41598-018-19418-7

**Published:** 2018-01-17

**Authors:** Eli Carlos Martiniano, Milana Drumond Ramos Santana, Érico Luiz Damasceno Barros, Maria do Socorro da Silva, David Matthew Garner, Luiz Carlos de Abreu, Vitor E. Valenti

**Affiliations:** 1 Núcleo de Estudos em Ciências Fisiológicas e Farmacêuticas, Faculdade de Juazeiro do Norte, Juazeiro do Norte, CE Brazil; 2 Laboratório de Delineamento de Estudos e Escrita Científica, Faculdade de Medicina do ABC, Santo André, SP Brazil; 3 Cardiorespiratory Research Group, Department of Biological and Medical Sciences, Faculty of Health and Life Sciences, Oxford Brookes University, Gipsy Lane, Oxford, OX3 0BP United Kingdom; 4 Centro de Estudos do Sistema Nervoso Autônomo (CESNA), Departamento de Fonoaudiologia, Faculdade de Filosofia e Ciências, UNESP, Marília, SP Brazil

## Abstract

Music can improve the efficiency of medical treatment when correctly associated with drug action, reducing risk factors involving deteriorating cardiac function. We evaluated the effect of musical auditory stimulus associated with anti-hypertensive medication on heart rate (HR) autonomic control in hypertensive subjects. We evaluated 37 well-controlled hypertensive patients designated for anti-hypertensive medication. Heart rate variability (HRV) was calculated from the HR monitor recordings of two different, randomly sorted protocols (control and music) on two separate days. Patients were examined in a resting condition 10 minutes before medication and 20 minutes, 40 minutes and 60 minutes after oral medication. Music was played throughout the 60 minutes after medication with the same intensity for all subjects in the music protocol. We noted analogous response of systolic and diastolic arterial pressure in both protocols. HR decreased 60 minutes after medication in the music protocol while it remained unchanged in the control protocol. The effects of anti-hypertensive medication on SDNN (Standard deviation of all normal RR intervals), LF (low frequency, nu), HF (high frequency, nu) and alpha-1 scale were more intense in the music protocol. In conclusion, musical auditory stimulus increased HR autonomic responses to anti-hypertensive medication in well-controlled hypertensive subjects.

## Introduction

Recently, adaptations in life-style have gained attention as a primary preventive of hypertension^1–3^. Music therapy is therefore a complementary intervention currently being investigated in cardiovascular physiology^4,5^ and hypertension treatments^6^.

A recent systematic meta-analysis investigated the effects of music therapy on blood pressure in hypertensive patients. The review selected references based on population, comparison, intervention, outcome and study type fundamentals to define the eligibility criteria. Only three studies achieved their rigorous inclusion conditions. The samples located in the study references ranged between 30 and 60 subjects with a patient mean age of 60 to 93 years. The review indicated that music therapy had significant positive effects on systolic arterial pressure (SAP) (−6.58 mmHg; 95% CI, −9.38 to −3.79 mmHg; p < 0.0001), but no significant statistical influence on diastolic arterial pressure (DAP) (−1.76 mmHg; 95% CI: −5.61 to 2.09 mmHg; p = 0.37) in hypertensive subjects^6^.

However, another systematic review did not support these findings. Kühlmann *et al*.^7^ followed the PRISMA guidelines and examined publications that evaluated the effects of music on arterial pressure in hypertensive patients through PubMed, Medline, Cochrane Central, Embase, Web of Science and Google Scholar. They found 10 studies among the preliminary 1689 references that satisfied the inclusion criteria. Yet the randomized controlled trials selected in the review reported a reduction in SAP from 144 mmHg to 134 mmHg and a decrease in DAP from 84 mmHg to 78 mmHg. No statistical significance was achieved.

The acute effects of music on cardiovascular parameters have been investigated in this context. Vlachopoulos *et al*.^8^ evaluated the short-term effect of music on arterial stiffness and wave reflections in healthy humans. They reported that classical and rock music reduced aortic stiffness and wave reflections.

Vanderlei *et al*.^9^ assessed cardiac autonomic function through heart rate (HR) variability (HRV), a non-invasive method that studies vagal and sympathetic influence on HR^10^ in healthy young women during exposure to the musical genres of baroque classical music and heavy-metal. In order to avoid the influence of sex hormones, the authors selected only females. The heavy metal music used was “Heavy Metal Universe” from Gamma Ray, which is based on an excitatory rhythm composed of a lead voice, guitar and bass. The classical music applied was the Canon in D by Johann Pachelbel, which is originally scored for three violins and basso continuo, and paired with a gigue. Both movements are in D major. It combines the forms of canon and ground bass. Three voices are engaged in canon, while a fourth voice and the basso continuo play an independent ground bass. The authors reported that heavy-metal music reduced parasympathetic control of HR while no significant change was achieved for classical music when applying nonlinear techniques for assessment. This was possibly due to the excitatory responses related to the heavy metal music style.

Roque *et al*.^11^ investigated the effects on healthy women of listening to baroque classical music and found no significant effect on linear indices of HRV. Also, it was conveyed that baroque classical music acutely decreased global modulation of HR through geometric HRV analysis. This effect was due to the sound intensity of the music^12^.

Accordingly, subjects of either gender diagnosed with irreversible pulpitis or pulp necrosis of the upper front teeth were exposed to music during endodontic treatment^13^. The subjects were randomly divided into two groups: one group submitted to surgical endodontic treatment without music, the other submitted to endodontic treatment while being exposed to music throughout the surgical procedures. The authors documented that although salivary cortisol levels were unaffected by music, music acutely attenuated HR autonomic responses during surgical treatment.

The studies mentioned above encourage us to investigate the association of music with cardiovascular interventions. Although earlier studies have already evaluated the acute effects of music on autonomic HR control, it is unclear whether music might influence the effect of medication on HRV, SAP and DAP. This information might assist clinicians in improving new pharmacological interventions for hypertension. With this in mind, we evaluated the acute effects of musical auditory stimulus associated with anti-hypertensive medication on cardiovascular variables in hypertensive subjects.

## Results

In the resting state, there was no significant difference between the control and music protocols regarding SAP (p = 0.25), DAP (p = 0.53) and LF/HF (p = 0.58). Reduced values of resting HR (p = 0.0014, Cohen’s d = 0.69, medium effect size), LF (nu) (p = 0.04, Cohen’s d = 0.39, small effect size) and alpha-1 (p = 0.0006, Cohen’s d = 0.75, medium effect size) were observed in the music protocol. Resting SDNN (p = 0.032, Cohen’s d = 0.41, small effect size), RMSSD (p = 0.02, Cohen’s d = 0.46, small effect size), pNN50 (p = 0.0.1, Cohen’s d = 0.56, medium effect size) and HF (nu) (p = 0.04, Cohen’s d = 0.39, small effect size) indices were higher in the music protocol.

Although the patients were clinically diagnosed hypertensive, only five patients had SAP ≥ 140 mmHg (maximum: 140 mmHg) and DAP ≥ 90 mmHg (maximum: 95 mmHg) because they were undergoing anti-hypertensive therapy.

**Figure 1:**
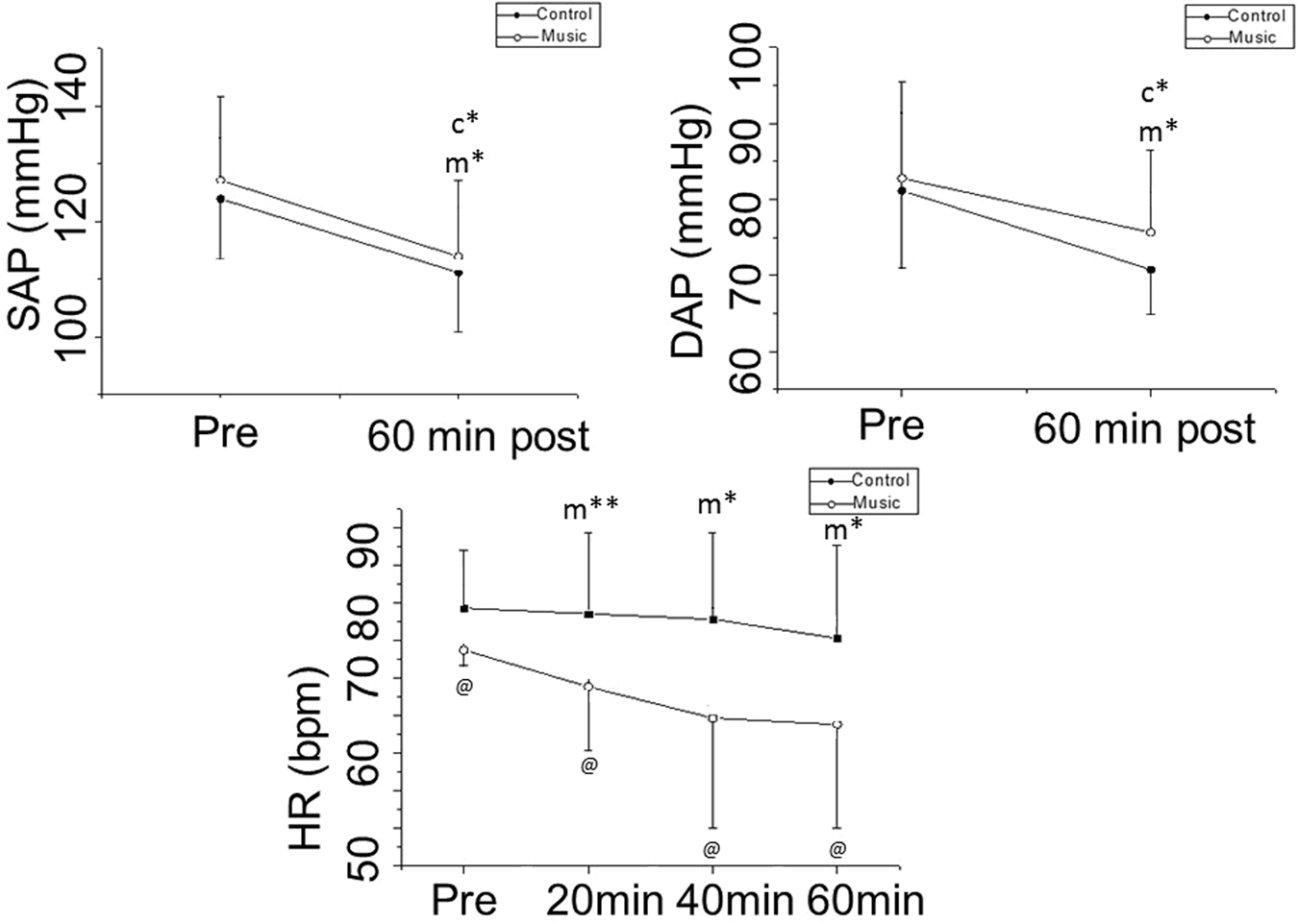
Systolic (SAP) and diastolic arterial pressure (DAP) and heart rate (HR) before and after medication in the music and control protocols. ^m*^p < 0.05 vs. Pre- for music protocol; ^m**^p < 0.05 vs. 60 min for music protocol; ^c*^p < 0.05 vs. Pre- for control protocol; ^@^p < 0.05 Control vs. Music protocol; Pre: Before medication.

With regard to the SAP and DAP responses to music after medication, we documented similar responses of SAP (control protocol: Cohen’s d = 1.17, large effect size; music protocol: Cohen’s d = 0.95, large effect size) and DAP (control protocol: Cohen’s d = 1.01, large effect size; music protocol: Cohen’s d = 0.6, medium effect size) in both procedures. SAP and DAP decreased after medication in the music and control protocols during the 60 minutes following medication (Fig. 1).

With the music protocol, HR decreased during minutes (pre-vs. 40 min – Cohen’s d: 0.75, medium effect size) and 60 minutes after medication (pre-vs. 60 min – Cohen’s d: 0.86, medium effect size) compared to before medication and reduced for 20 minutes compared to 60 minutes after medication (20 min vs. 60 min – Cohen’s d: 0.44, small effect size). With the control protocol, HR was unaffected after medication (Fig. 1).

There was a significant protocol interaction (control protocol vs. music protocol) for HR; we found values reduced in the music protocol by 20 minutes (Cohen’s d = 1, large effect size), 40 minutes (Cohen’s d = 1, large effect size) and 60 minutes (Cohen’s d = 1, large effect size) after medication equated to the control protocol at the same instants (Fig. 1).

**Figure 2:**
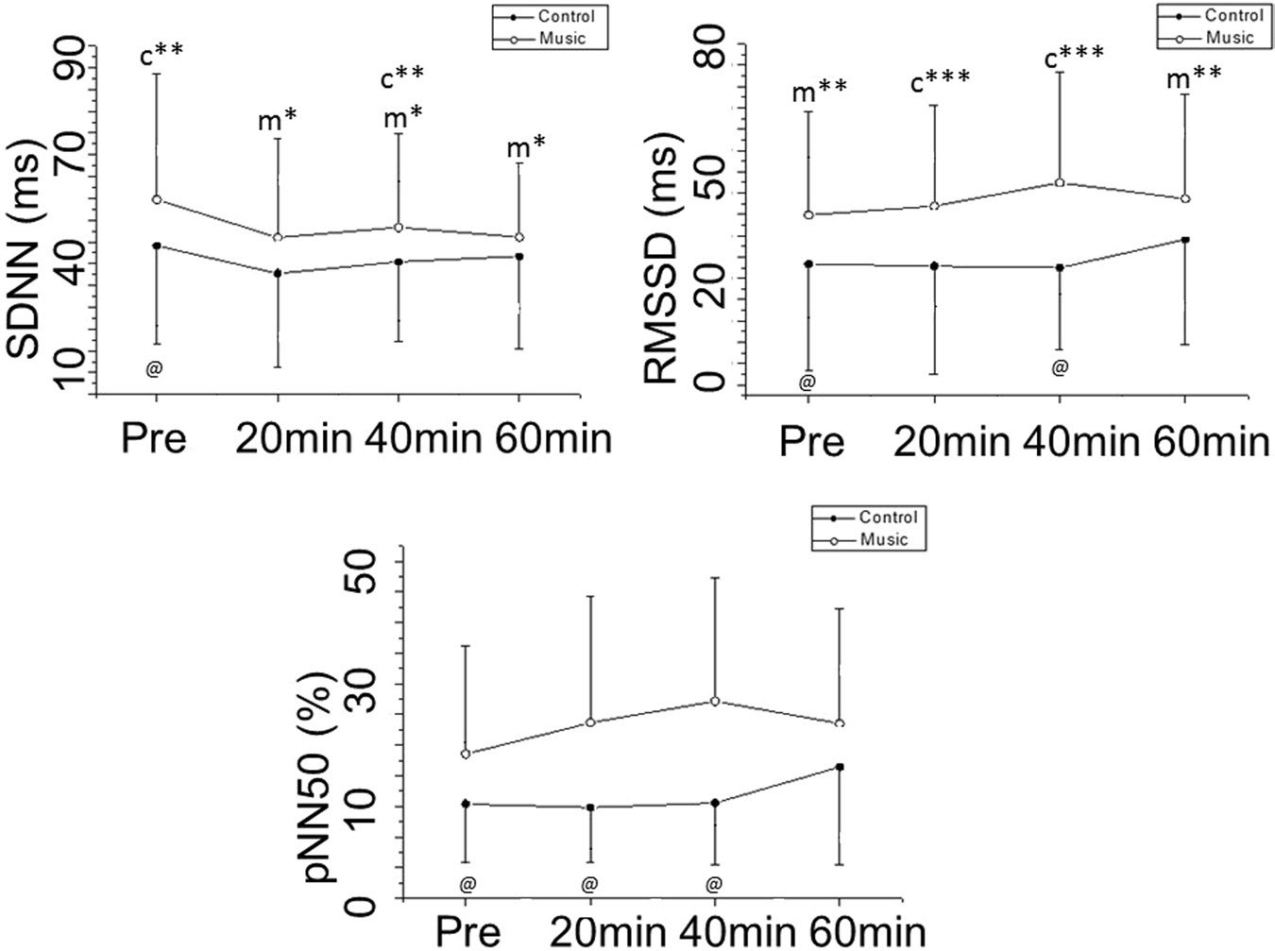
Time domain indices of HRV before and after medication in the control and music protocols. Pre: Before medication; pNN50: the percentage of adjacent RR intervals with a difference of duration greater than 50 ms; RMSSD: root-mean square of differences between adjacent normal RR intervals in a time interval; ms: milliseconds; SDNN: Average standard deviation of normal RR intervals; ms: milliseconds. ^m*^p < 0.05 vs. Pre- for music protocol; ^c**^p < 0.05 vs. 20 min for control protocol; ^c***^p < 0.05 vs. 60 min for control protocol, ^m**^p < 0.05 vs. 40 min for music protocol; ^@^p < 0.05 Control vs. Music protocol.

With regard to the time domain analysis for HRV, SDNN was reduced in the control protocol after 20 minutes of medication compared to control (pre-vs. 20 min, Cohen’s d: 0.53, medium effect size) and 40 minutes compared to 20 minutes after medication (20 min vs. 40 min, Cohen’s d: 0.28, small effect size). In the music protocol SDNN was lessened 20 minutes (pre-vs. 20 min, Cohen’s d: 0.33, small effect size), 40 minutes (pre-vs. 40 min, Cohen’s d: 0.25, small effect size) and 60 minutes (pre-vs. 60 min, Cohen’s d: 0.36, small effect size) after medication compared to before medication. RMSSD increased 60 minutes compared to 20 minutes (20 min vs. 60 min: Cohen’s d: 0.96, large effect size) and 40 minutes after medication (40 min vs. 60 min: Cohen’s d: 0.54, medium effect size) in the control protocol. In the music protocol, RMSSD increased 40 minutes after medication compared to before (pre-vs. 40 min: Cohen’s d: 0.3, small effect size) and 60 minutes after medication (40 min vs. 60 min: Cohen’s d: 0.14, below the threshold) (Fig. 2).

We observed the interaction of the procedure (control protocol vs. music protocol) for RMSSD and pNN50. RMSSD was elevated 40 minutes after medication in the music protocol (Cohen’s d: 2.35, large effect size) and pNN50 was higher 20 after minutes (Cohen’s d: 0.87, medium effect size) and 40 minutes after medication in the music protocol (Cohen’s d: 1.04, large effect size) compared to the control protocol at the same times (Fig. 2).

**Figure 3:**
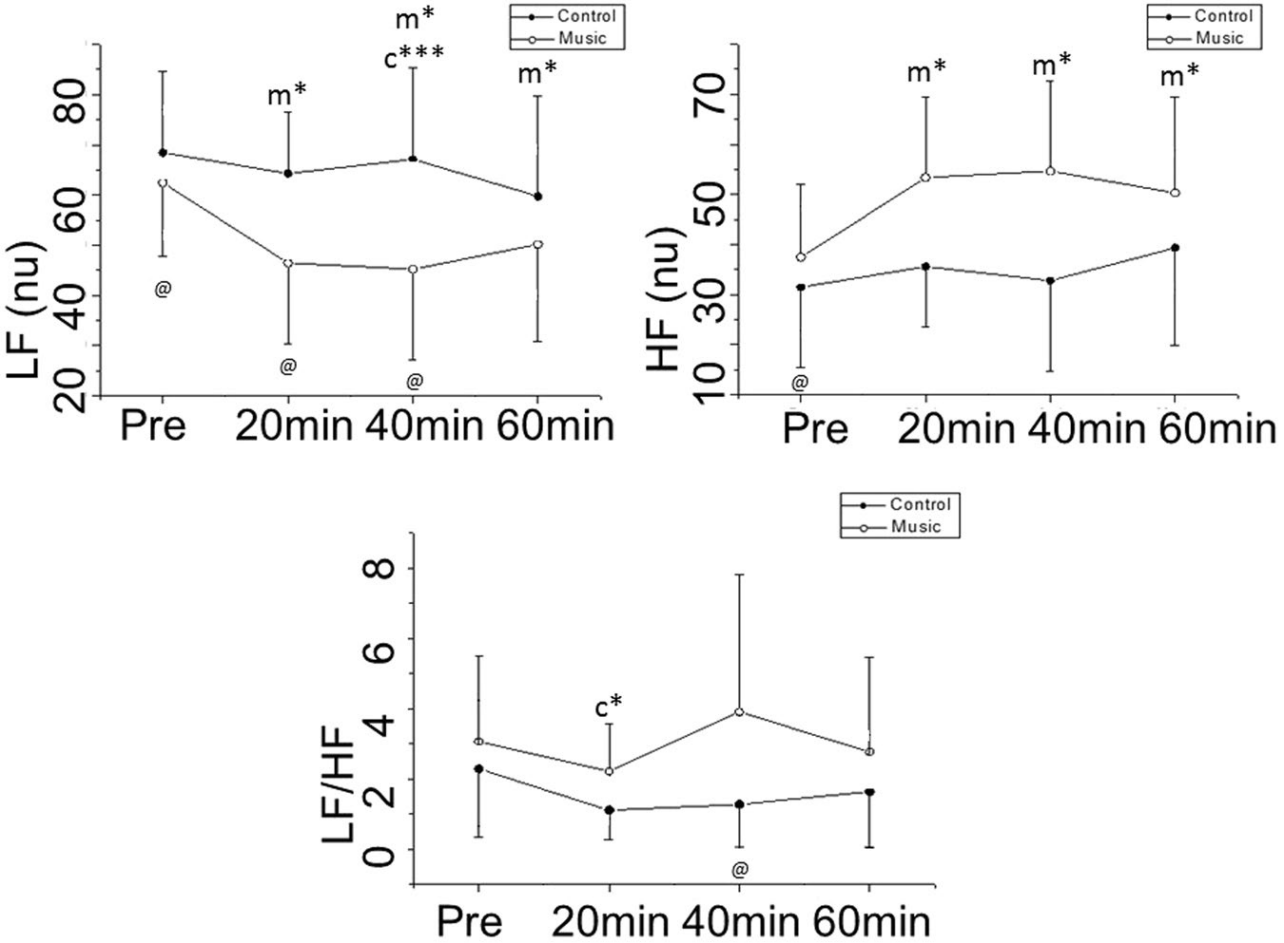
Frequency domain indices of HRV in normalized units before and after medication in the control and music protocols. Pre: Before medication; LF: low frequency; HF: high frequency; LF/HF: low frequency/high frequency ratio; ms: milliseconds. ^m*^p < 0.05 vs. Pre- for music protocol; ^c*^p < 0.05 vs. Pre- for control protocol; ^c***^p < 0.05 vs. 60 min for control protocol; ^@^p < 0.05 Control vs. Music protocol.

**Figure 4:**
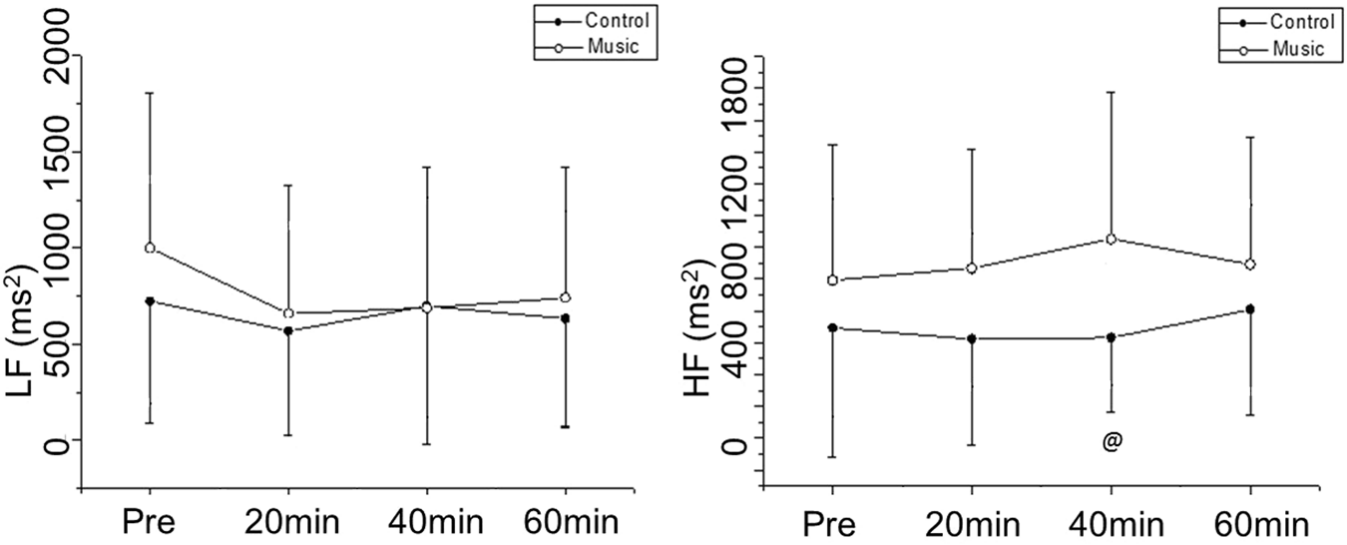
Frequency domain indices of HRV in absolute units before and after medication in the control and music protocols. Pre: Before medication; LF: low frequency; HF: high frequency; LF/HF: low frequency/high frequency ratio; ms: milliseconds; ^@^p < 0.05 Control vs. Music protocol.

Spectral analysis of HRV is shown in Figs 3 and 4. LF (nu) declined 60 minutes after medication compared to 40 minutes after medication (60 min vs. 40 min, Cohen’s d: 0.38, small effect size) in the control protocol while it decreased 20 minutes (pre-vs. 20 min, Cohen’s d: 1.03, large effect size), 40 minutes (pre-vs. 40 min, Cohen’s d: 1.04, large effect size) and 60 minutes (pre-vs. 60 min, Cohen’s d: 0.71, medium effect size) after medication compared to before medication in the music protocol.

HF (nu) increased 20 minutes (pre-vs. 20 min, Cohen’s d: 1.03, large effect size), 40 minutes (pre-vs. 40 min, Cohen’s d: 1.04, large effect size) and 60 minutes after medication compared to before medication (pre-vs. 60 min, Cohen’s d: 0.75, medium effect size) in the music protocol whereas no significant changes were revealed in the control protocol regarding HF (nu).

The LF/HF ratio was reduced 20 minutes after medication compared to before medication (pre-vs. 20 min, Cohen’s d: 0.79, medium effect size) in the control protocol (Fig. 3).

There was a procedure interaction (control protocol vs. music protocol) for LF (nu) and LF/HF. The LF band decreased 20 minutes (Cohen’s d: 1.24, large effect size) and 40 minutes (Cohen’s d: 1.21, large effect size) after medication in the music protocol compared to the control protocol at the same time. The LF/HF ratio was higher 40 minutes (Cohen’s d: 0.83, medium effect size) after medication in the music protocol compared to the control protocol at the same time (Fig. 3).

Regarding frequency domain indices in absolute units, there was no moment of interaction for LF. Equally, we noted a procedure interaction (control protocol vs. music protocol) for the HF band. HF at 40 minutes in the music protocol was increased compared to 40 minutes after medication in the control protocol at the same time (Cohen’s d: 0.85, medium effect size) (Fig. 4).

**Figure 5:**
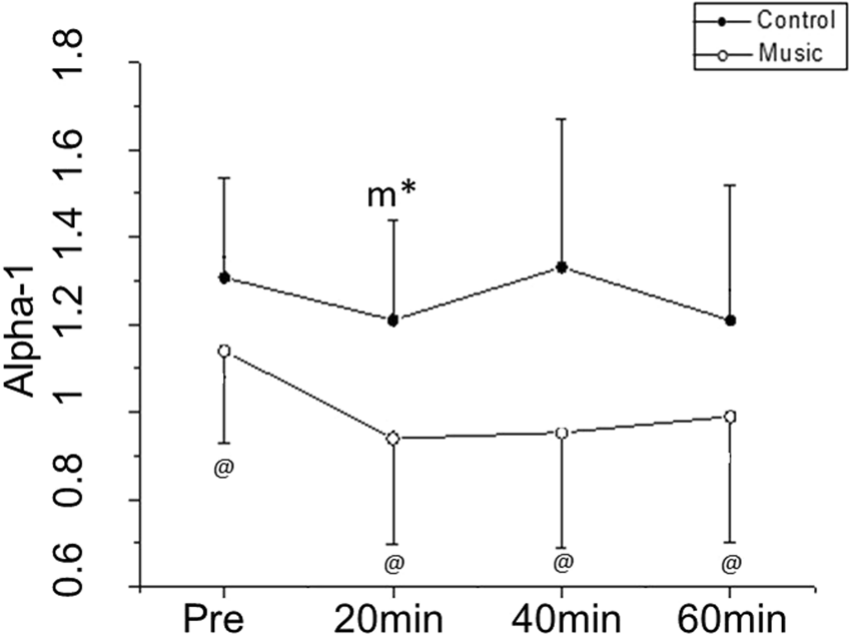
Fractal correlation property of HRV before and after medication in the control and music protocols. Pre: Before medication; ^m*^p < 0.05 vs. Pre- for music protocol; ^@^p < 0.05 Control vs. Music protocol.

Nonlinear analysis of HRV by Detrended Fluctuation Analysis (DFA) indicated that alpha-1 decreased 20 minutes (Cohen’s d: 0.88, medium effect size) after medication compared to before medication in the music protocol whereas no change was noted in the control protocol (Fig. 5).

We noticed a procedure interaction (control protocol vs. music protocol) for the alpha-1 scale, which was reduced 20 minutes (Cohen’s d: 3.9, large effect size), 40 minutes (Cohen’s d: 1.23, large effect size) and 60 minutes (Cohen’s d: 2.91, large effect size) after medication in the music protocol compared to the control protocol at the same times (Fig. 5).

## Discussion

Hypertension is increasingly regarded as a widespread global disease. As such, it is looked upon as an important factor in the development of conditions aggravating the cardiovascular system, marking it as an important public health problem^14^. According to Santos *et al*.^1^ there are several ways to establish an effective treatment but controlling blood pressure alone is inadequate. Music therapy has been investigated as a possible contributor to the treatment of hypertension^6^.

We set out to evaluate the acute effects of musical auditory stimulus associated with anti-hypertensive medication on HRV in well-controlled hypertensive patients. Of chief importance, we detected that music acutely intensified the effect of anti-hypertensive medication on HRV. The effect size calculations for spectral analysis and DFA upheld the added intense response of HR dynamics when medication was linked with music, equated to medication administrated in the absence of music.

The period of the medication response changes in relation to the type of treatment and its configuration. In most cases the effects begin to appear around 15 to 20 minutes after administration and approaches the optimum therapeutically around 60 minutes later^15^.

All medical drugs in this study generate significant changes in vagal control of HR as stated in the research literature^16–20^. Based on our results, anti-hypertensive medication presented more intense effects on HR when associated with music, even taking into account that resting HR was reduced during the music protocol, thus reinforcing the influence of music on parasympathetic responses induced by such medications.

Similarly, the influence of music on changes in HRV induced by medication were observed in the HF band, indicating the parasympathetic regulation of HR^10^. Spectral analysis of HRV revealed that HF significantly increased in normalized units for the entire 60 minutes after medication administration associated with music, while it remained unchanged when the medication was administered alone. Statistical analysis via protocol interaction demonstrated that HF in absolute units (ms^2^) significantly increased 40 minutes after medication in the music protocol compared to the control protocol for the same period. This reinforces the view that music enhances the therapeutic effects anti-hypertensive medication had on HRV.

Furthermore, we detected increased RMSSD values 40 minutes after medication in the music protocol compared to the control protocol at the same time. Also, higher pNN50 values were noted in the music protocol 20 minutes and 40 minutes after medication compared to the control protocol at the same times, suggesting that music augmented the pharmacological effects.

The influence of music on HRV responses to anti-hypertensive medication is supported by the large effect size found in the HF band (nu) for moment interaction, which can also be observed in pNN50 and RMSSD for protocol interaction.

Previous studies verified the effect of music on vagal HR regulation^4^. Nakamura *et al*.^5^ studied gastric vagal nerve activity in urethane-anesthetized rats during exposure to a relaxant music. The investigators observed elevated parasympathetic activity when rats were exposed to relaxant music (Schumann: Traeumerei). But during white noise there was no significant change in vagal activity. Through expression of c-Fos protein, it was revealed that this mechanism was dependent on the auditory cortex.

An additional study investigated the effect of Mozart’s music on HRV in 64 Taiwanese children whose ages ranged from 7 ± 3 years old (extending from 2 years 11 months to 15 years 4 months)^21^. The researchers determined that changes in parasympathetic modulation of HR is involved in the reduction of epileptiform discharges during exposure to Mozart’s music.

We therefore support the view that the intensification of HR responses to anti-hypertensive medication in the music protocol is because music can intensify the vagal activity response induced by such pharmacological treatments. We accept that music could activate the parasympathetic system, as stated by Nakamura *et al*.^5^ causing an increase in gastrointestinal activity, thus accelerating the absorption of anti-hypertensive medication and intensifying the effects on HRV.

However, previous studies have reported conflicting results regarding the reduction of HR vagal regulation induced by music. Roque *et al*.^11^ detected that young adult women exposed to classical baroque music (Pachelbel: Canon in D) delivered no change in parasympathetic regulation of HR. The same judgement was supported by da Silva *et al*.^22^. Then again, Ferreira *et al*.^23^ stated increased global modulation of HR followed exposure to the same classical baroque music.

A methodological factor that could explain the opposing results concerning musical effects on HR autonomic control is associated with the musical genre applied. The studies mentioned above prescribed different music compared to the music evaluated in our investigation. Under these circumstances, musical genre is a critical consideration when investigating the influence music has on HRV^24^.

Similarly, we completed nonlinear dynamic analysis of HR after anti-hypertensive medication alone and then accompanying music. The research literature reports that adrenergic blockers increase the nonlinear behavior of HR dynamics, which is advantageous^25^.

Based on our statistics, alpha-1 scale was unstable between 1.2 and 1.4 in the control protocol, while it decreased and remained close to 1.0 in the music protocol. Cohen’s d displayed medium effect size for moment interaction in the music protocol and large effect size for protocol interaction. The most complex structure based on fractal fluctuations is alpha equal to 1.0^26–29^. Thus, our results demonstrate that anti-hypertensive medication increases the nonlinear dynamics of HR. This response was exaggerated by the musical stimulus.

Our study contains further points which need highlighting. Auditory equivalent levels were not measured, therefore it is impossible to detect any effect related to sound intensity. Musical empathy scale was not applied since such a questionnaire was not validated in the subjects’ language. We did not choose specific pharmacological agents, since the medications directed encompassed diuretics, calcium channel blockers and beta-blockers. This would have delivered essential information regarding which hormone or neurotransmitter is involved in the HRV responses to medication associated with the music applied. There was a significant age range among the sample, 28 to 83 years old, however, the sample investigated is characteristic of the clinical population.

Different periods of medication response are a constraint in our study. We attempted to minimize this bias by matching the groups according to the length of time they had been taking medication, specifically patients were under anti-hypertensive treatment for between six months and one year.

One clarification of the positive acute effects of music on anti-hypertensive medication is that the musical sequence was repeated four times. A recent study applied repeated songs and evaluated HRV in subjects under stressful conditions. Santana *et al*.^13^ stated that it positively influenced HRV during endodontic treatment. In this context, we suggest that the repeated music sequences used in our study may become familiar to the patient and elicit a calming effect.

Here, we investigated a specific group of well-controlled hypertensive patients. We strongly encourage additional investigation under different pathological conditions to perform further experiments to detect whether music has a significant influence on pharmacological action.

It has recently been suggested that treatment of hypertension should be based on life-style behaviors including physical activity and/or giving up smoking^3^. In this context, complementary therapies applied to assist hypertensive treatment may further decrease dependence on pharmacological intervention. This is always beneficial.

## Conclusion

Musical auditory stimulus intensified HR autonomic responses to anti-hypertensive medication in well-controlled hypertensive subjects.

## Methods

### Study population

We enrolled 37 subjects clinically diagnosed as hypertensive (14 men; 64.6 ± 12.54 years old, 1.64 ± 0.11 m, 64.41 ± 11.88 kg, 23.89 ± 2.95 kg/m^2^) with controlled blood pressure (systolic arterial pressure (SAP) 123.87 ± 10.5 [105–140] mmHg; diastolic arterial pressure (DAP) 80.25 ± 7.5 [60–90] mmHg) who had been undergoing anti-hypertensive treatment for between six months and a year. Medications taken by subjects included Hydrochlorothiazide 25 mg (HCTZ) (7), HCTZ 25 mg/Captopril 25 mg (6), HCTZ 25 mg/Losartan 50 mg (2), Enalapril 5 mg (4), Captopril 25 mg (7), Atenolol 25 mg (4), Anlodipine 2,5 mg (4) and Losartan 25 mg (3).

All subjects gave their confidential informed written consent to participate in the research study. The Ethics Committee in Research of the Faculty of Juazeiro do Norte approved all study procedures (No. 1.458.187) and were in accordance with National Health Resolution 466/2012.

We did not include subjects with cardiorespiratory, neurological and endocrine disorders associated with hypertension, smokers or subjects suffering from related diseases that did not permit performance of the protocols or subjects under treatment but with no anti-hypertensive medication. Athletes and physically active subjects were also excluded in order to homogenize the population because the literature indicated that physical activity influences HRV^30^.

### HRV analysis

HRV was recorded using the Polar RS800CX device (Polar Electro Oy, Kempele, Finland) previously validated to identify beat-to-beat HR^31^ and assessed from stationary sequences manually selected, which were defined by the stability of the mean duration and the variance of the RR interval duration. Stationary sequences matched the periods with the lowest changes in HRV over time. These RR interval sequences are related to periods with homogeneous HRV and resembled periods with limited environmental influences. We examined a 10-minute time window for HRV analysis and cubic spline interpolation of RR intervals was standardized to 4 Hz. Details of HRV analysis have been described previously^32^.

The Polar transmitter identifies all RR intervals through electrical signals and the data recorded transmits the signal to the computer with a tool through Bluetooth.

We performed digital filtering to eliminate artifacts. The digital filtering in the software was based on an algorithm that calculated median and moving average. The algorithm in the software calculated several more matching RR intervals to substitute the detected errors. Before creating the preview curve, the algorithm checks the difference between consecutive RR intervals and makes a series of corrected values (typically 2–4 intervals) to follow an indistinguishable discrepant coefficient. The algorithm accurately maintains the total period of HR recording and the number of RR intervals is precisely the same as the elapsed period. We standardized six bpm for the minimum protection zone (http://support.polar.com/en/support/tips/How_R-R_Data_is_Filtered).

After we performed digital filtering followed by manual filtering for the elimination of artifacts, we selected 256 stable RR intervals. We included series with less than 5% artefact^32^.

### HRV Time and Frequency Domain Indices

We evaluated the following time domain indices of HRV: RMSSD – is the root-mean square of differences between consecutive RR intervals; SDNN – standard deviation of RR intervals and; pNN50 – percentage of adjacent RR intervals with a difference higher than 50 ms^33^ (Table 1).

**Table 1:** Reference table relatively to the HRV acronyms.

Variable	Full description
pNN50	The percentage of adjacent RR intervals with a difference of duration greater than 50 ms
RMSSD	Root-mean square of differences between adjacent normal RR intervals in a time interval
SDNN	Average standard deviation of normal RR intervals
LF	Low frequency
HF	High frequency

In relation to the spectral analysis of HRV analysis, the RR intervals underwent mathematical processing, generating a tachogram that showed the variation of RR intervals as a time function. The tachogram has a signal that ranges in time and was processed by the mathematical Fast Fourier Transform algorithm^10^. The Welch periodogram method based on Fast Fourier Transform was used with a window overlap of 50% and a window width of 256 seconds.

High frequency (HF- ranging from 0.15 to 0.4 Hz) and low frequency (LF- ranging from 0.04 to 0.15 Hz) spectral parameters were selected in normalized (nu) and absolute units (ms^2^). The ratio between LF and HF in absolute values (LF/HF) corresponds to the relative value of each spectral parameter related to total power minus very and ultra low frequency components^10^ (Table 1).

We used the Kubios HRV^®^ v.1.1 for Windows software to measure the linear indices^33^.

### Nonlinear HRV analysis

Nonlinear analysis was undertaken by DFA, alpha-1 determined the short term (4–12 beats) correlations between successive heart beats. Details regarding DFA were previously described^26–28^.

We considered α = 0.05 as no correlation (the signal consists of white noise); when α = 1.5 we considered random walk (Brownian motion); and when 0.5 < α < 1.5 we considered positive correlations. If alpha is close to 1.0 it indicates a more complex (non-linear) system; if it reaches values above 1.0 the system tends to be less complex and linear^26–28^.

### Protocols

Data collection was undertaken in the same room for all subjects; the temperature was controlled between 21 °C and 25 °C and the relative humidity was between 40% and 60%. Subjects were told not to ingest alcohol, caffeine or other substances likely to stimulate the autonomic nervous system for 24 hours before the evaluation, maintaining an empty bladder with only a light meal 2 to 3 hours before collection of the experimental data. Data sets were obtained between 8:00 and 16:00 to standardize circadian influences.

HRV was analyzed seated at rest under spontaneous breathing in the following periods: 1) control protocol - the 10-minute period before the medication; 2) 20 minutes after oral medication; 3) 40 minutes after oral medication and; 4) 60 minutes after oral medication. HRV was analyzed in the final 5 minutes of each period.

Each subject was submitted to two procedures performed on two random days.

For the music protocol, after 10 minutes of rest under spontaneous breathing, the volunteers were submitted to the following music stimulus via an earphone: 1-Someone like you (instrumental piano) – by Adele; 2-Airstream – by Electra; 3-Hello (instrumental piano) – by Adele; 4-Amazing grace [my chains are gone] [instrumental] by Chris Tomlin and 5-Watermark by Enya. The music sequence was repeated 4 times. Music was played throughout the 60 minutes after medication with the same volume for all subjects. The volunteers were told to keep at rest and avoid conversation during the protocol.

In the control protocol, the subjects continued similarly with the earphone turned off.

**Figure 6:**
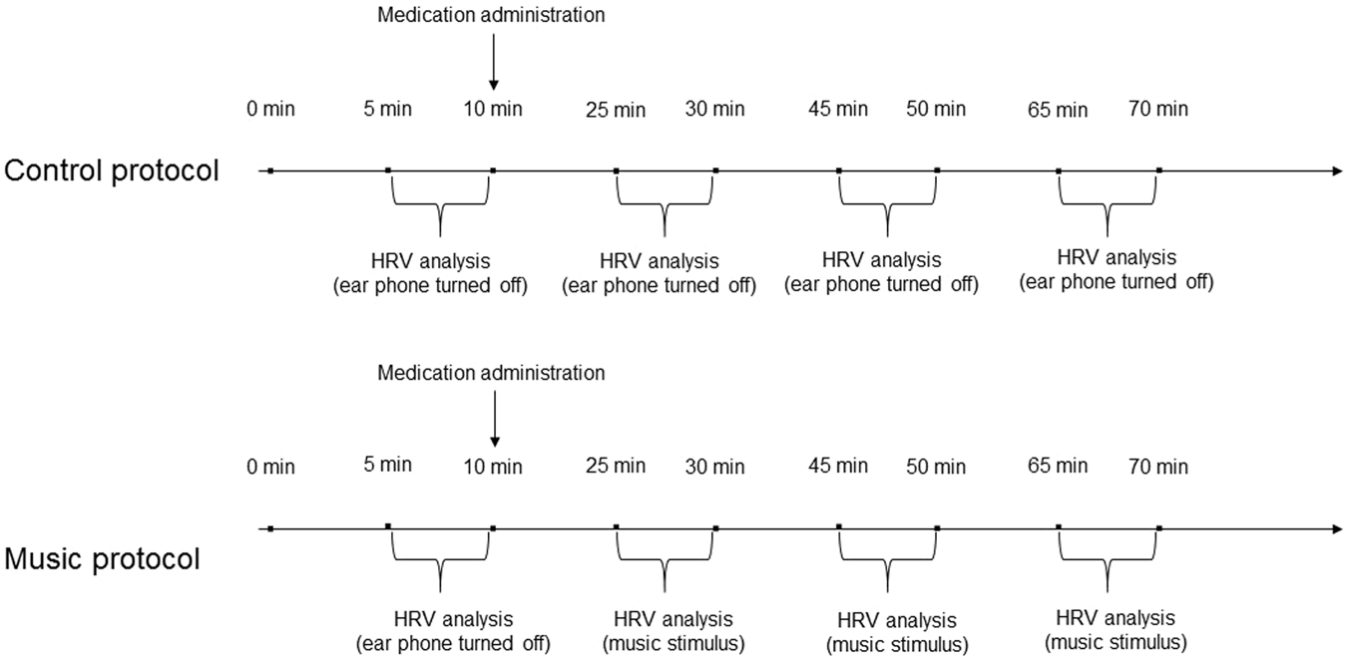
Scheme of the experimental design.

Figure 6 presents a scheme of the experimental design.

### Statistical analysis

The sample size calculation was based on the rest RMSSD index according to the study by Moreno *et al*.^34^. We assumed a magnitude of the difference for 11 ms, and considered a standard deviation of 16.2 ms, beta risk of 80% and alpha risk of 5%. A minimum of 18 subjects were attained.

The Shapiro-Wilk goodness-of-fit test verified the normal Gaussian distribution of the data (z value > 1.0).

Comparisons of HRV values between procedures (control and music) and times (rest vs. recovery periods) were carried out through the analysis of variance technique to model repeated measures on the two factors scheme. Data from repeated measurements were verified for evaluation of sphericity using the Mauchly test. Greenhouse-Geisser correction was applied when the sphericity was violated.

To consider the two phases (rest vs. recovery periods) we applied the Bonferroni post-test for parametric distribution or Dunn’s post-test for non-parametric distribution. We considered statistical significance for p < 0.05.

To compute the magnitude of difference between groups and between two time-periods, the effect size was calculated using Cohen’s *d* for significant differences. Large effect size was considered for Cohen’s d ≥ 0.9, medium for Cohen’s d between 0.9 and 0.5 and small for Cohen’s d between 0.5 and 0.25^35^.
